# ND‐AMD: A Web‐Based Database for Animal Models of Neurological Disease With Analysis Tools

**DOI:** 10.1111/cns.70411

**Published:** 2025-05-09

**Authors:** Yue Wu, Lu Li, Yi‐Tong Li, Lei Zhang, Shuang Gong, Yang Zhang, Jue Wang, Ling Zhang, Qi Kong

**Affiliations:** ^1^ Institute of Laboratory Animal Sciences, CAMS & PUMC, National Human Diseases Animal Model Resource Center, National Center of Technology Innovation for Animal Model, NHC Key Laboratory of Comparative Medicine, State Key Laboratory of Respiratory Health and Multimorbidity, Key Laboratory of Pathogen Infection Prevention and Control (Peking Union Medical College) Ministry of Education Beijing China; ^2^ Nutshell Therapeutics (Shanghai) co., Ltd Shanghai China

**Keywords:** analysis tools, animal models, behavioral test, bioinformatics, database, degenerative diseases, neuroscience

## Abstract

**Background:**

Research on animal models of neurological diseases has primarily focused on understanding pathogenic mechanisms, advacing diagnostic strateggies, developing pharmacotherapies, and exploring preventive interventions. To facilitate comprehensive and systematic studies in this filed, we have developed the *Neurological Disease Animal Model Database* (ND‐AMD), accessible at https://www.uc‐med.net/NDAMD. This database is signed around the central theme of “Big Data ‐ Neurological Diseases ‐ Animal Models ‐ Mechanism Research,” integrating large‐scale, multi‐dimensional, and multi‐scale data to facilitate in‐depth analyses. ND‐AMD serves as a resource for panoramic studies, enabling comparative and mechanistic research across diverse experimental conditions, species, and disease models.

**Method:**

Data were systematically retrieved from PubMed, Web of Science, and other relevant databases using Boolean search strategies with standardized MeSH terms and keywords. The collected data were curated and integrated into a structured SQL‐based framework, ensuring consistency through automated validation checks and manual verification. Heterogeneity and sensitivity analyses were conducted using Cochran's Q test and the *I* ^2^ statistic to assess variability across studies. Statistical workflows were implemented in Python (SciPy, Pandas, NumPy) to support multi‐scale data integration, trend analysis, and model validation. Additionally, a text co‐occurrence network analysis was performed using Natural Language Processing (TF‐IDF and word embeddings) to identify key conceptual linkages and semantic structures across studies.

**Results:**

ND‐AMD integrates data from 483 animal models of neurological diseases, covering eight disease categories, 21 specific diseases, 13 species, and 152 strains. The database provides a comprehensive repository of experimental and phenotypic data, covering behavioral, physiological, biochemical, molecular pathology, immunological, and imaging characteristics. Additionally, it incorporates application‐oriented data, such as drug evaluation outcomes. To enhance data accessibility and facilitate in‐depth analysis, ND‐AMD features three custom‐developed online tools: *Model Frequency Analysis, Comparative Phenotypic Analysis, and Bibliometric Analysis,* enabling systematic comparison and trend identification across models and experimental conditions.

**Conclusions:**

The centralized feature of ND‐AMD enables comparative analysis across different animal models, strains, and experimental conditions. It helps capture intricate interactions between biological systems at different levels, ranging from molecular mechanisms to cellular processes, neural networks, and behavioral outcomes. These models play a vital role as tools in replicating pathological conditions of neurological diseases. By offering users convenient, efficient, and intuitive access to data, ND‐AMD enables researchers to identify patterns, trends, and potential therapeutic targets that may not be apparent in individual studies.

## Graphical abstract

A standardized, authoritative, and highly influential database for animal models of neurological disease with model frequency, comparative phenotypic, and bibliometric analysis tools. This comprehensive resource encompasses 483 animal models across eight disease types, spanning 21 diseases, 13 species, and 152 strains with detailed model and phenotype information.

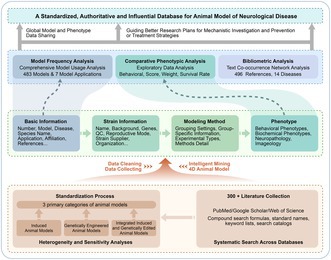

**Abbreviations**
6‐OHDA: 6‐hydroxydopamine
AD: Alzheimer's disease
ADHD/ASD: Attention deficit hyperactivity disorder's disease/Autism spectrum disorder's disease
ANCOVA: Analysis of covariance
ANOVA: Analysis of variance
APP: Amyloid precursor protein
Aβ: Amyloid‐beta
BD: bipolar disorder
CNS tumor: Central nervous system tumor
EDA: Exploratory data analysis
HD: Huntington's disease
HTT: Huntingtin
MAPT: Microtubule‐Associated Protein Tau
MPTP: 1‐methyl‐4‐phenyl‐1,2,3,6‐tetrahydropyridine
MRI: Magnetic resonance imaging
ND: Neurological diseases
ND‐AMD: Neurological disease animal model database
NLP: Natural language processing
PD: Parkinson's disease
PSEN1: Presenilin 1
SQL: Structured query language

## 1 Background

Neurological diseases (NDs) encompass a wide range of disorders characterized by structural and functional abnormalities in the nervous system, leading to impairments in cognitive, perceptual, behavioral, and emotional functions. These conditions range in severity from mild to debilitating, significantly impacting patients' quality of life and imposing a substantial burden on healthcare systems worldwide. Despite advancements in understanding the pathophysiology of NDs, many disorders remain poorly characterized, and effective treatments are still limited. This highlights the urgent need for innovative research strategies to unravel disease mechanisms and develop targeted therapies [1, 2, 3, 4].

Animal models have long been indispensable in neuroscience research, providing critical insights into the pathogenesis, progression, and comorbidities of neurological diseases. By replicating key aspects of human conditions, these models facilitate investigations into alterations in brain structure, function, and molecular pathways, enabling the identification of potential therapeutic targets, biomarkers, and diagnostic indicators [5, 6]. Additionally, they serve as essential platforms for preclinical drug testing, bridging the gap between fundamental research and clinical applications [7, 8].

Over the years, a diverse array of animal models has been established, predominantly in rodents (mice and rats), but also in non‐human primates, rabbits, and pigs. These models cover a wide range of neurological conditions, including neurodegenerative disorders, neuropsychiatric diseases, autoimmune diseases of the nervous system, and cerebrovascular disorders [9, 10, 11, 12]. However, the complexity and heterogeneity of these diseases necessitate large‐scale data integration, visualization, and comparative analysis to extract meaningful biological insights. Without a centralized approach, researchers face challenges in systematically comparing models, identifying shared pathological mechanisms, and leveraging cross‐species analyses for translational research.

Several existing databases such as Alzforum (The Alzheimer Research Forum Web site, https://www.alzforum.org), Alzdata database (http://www.alzdata.org/), AMP‐AD (Accelerating Medicines Partnership‐Alzheimer's Disease, https://www.nia.nih.gov/research/amp‐ad), The Jackson Laboratory Alzheimer's Disease Center (https://www.jax.org/explore‐by‐topic/neurodegenerative‐disease/alzheimers‐disease#), and MODEL‐AD (The Model Organism Development and Evaluation for Late‐onset Alzheimer's Disease, https://www.model‐ad.org) etc., primarily focus on neurodegenerative diseases and high‐throughput datasets. However, these databases have limitations in their coverage of animal models, particularly regarding phenotypic diversity and multi‐species comparisons [13, 14, 15, 16, 17].

To address these limitations, there is a critical need for a centralized and comprehensive database that integrates diverse animal models of neurological diseases, encompassing model development, phenotype analysis, key disease‐stage indicators, and application scopes. Such a resource should systematically capture spatiotemporal phenotypic data across different disease stages, offering a dynamic and multidimensional representation of human pathophysiology. Furthermore, the structured collection and sharing of fundamental, experimental, and phenotypic data—including behavioral, physiological, biochemical, molecular pathology, immunological, and imaging datasets—would allow researchers to uncover patterns, trends, and therapeutic targets that may not be apparent in individual studies. Integrating statistical methodologies and visualization tools would further enhance analytical capabilities, providing users with an efficient, intuitive, and scalable platform for conducting multi‐scale and multi‐dimensional analyses.

## 2 Materials and Methods

### 2.1 Literature Retrieval and Collection

Thorough identification of relevant literature is critical component of meta‐like‐analyses, as the validity of the findings relies heavily on the selection of included studies [18]. To ensure robustness and reliability, a comprehensive search strategy across multiple databases, including Google Scholar, PubMed, and Web of Science, was employed to retrieve pertinent literature [19]. This process involved the use of compound search formulas, combining terms such as “neurological disease name” with “animal model species” as keywords to systematically retrieve and download relevant literature. Additionally, standard diseases nomenclature was compiled to establish keyword lists and search catalogs, further refining the search process. Priority was given to recent high‐impact review articles to ensure the inclusion of influential studies. Literature collection was structured according to different disease categories, with the symbol “*” used for general retrieval encompassing synonymous terms for neurological diseases. To maintain rigor and relevance, several exclusion criteria were applied. We excluded non‐English literature, reviews, book chapters, conference papers, notes, editorials, commentaries, opinions, and case reports from our analysis. This stringent selection process ensured that only highquality, peer‐reviewed primary research studies were incorporated, thereby enhancing the reliability of the dataset.

Inclusion criteria were established to ensure the selection of high quality literature, prioritizing studies that provide comprehensive and transparent documentation of neurological animal models. Eligible studies were required to specify key aspects of model development, including detailed animal background information (species, strain, genetic modifications) and rigorously validated modeling methodologies, such as neurotoxin induction, genetic engineering, or surgical interventions with standardized protocols. Additionally, only studies with experimental designs, including appropriate control groups, standardized phenotyping protocols, and assessments of model reliability and validity, were considered [20]. Preference was given to literature that included detailed phenotype characterizations, particularly those emphasizing disease similarity and reproducibility in relation to human conditions (Table 1). Studies were excluded if they lacked essential methodological details (e.g., incomplete documentation of experimental protocols or results), employed non‐standardized approaches, or failed to provide sufficient data for reproducibility and comparative analysis.

**TABLE 1:** Inclusion criteria for literature selection.

Criterion	Description
Animal background	Species, strain, age, sex, and genetic modifications must be explicitly reported
Modeling method validity	Protocols must be validated in at least two independent studies or comply with established guidelines
Phenotype data	Must include behavioral, biochemical, pathological, or imaging data to allow cross‐study comparisons
Human disease relevance	Models must demonstrate face and/or construct validity in relation to human neurological diseases
Peer‐reviewed publication	Only studies published in peer‐reviewed journals were considered

A directory of retrieved and eligible literature was compiled, listing complete and unabridged literature titles along with corresponding PDF access links. Each entry indicated whether the full text was successfully downloaded (“Y”) or not (“N”). All downloaded literature (PDF format) was systematically gathered by a designated curator, with proper data backup procedures in place to ensure integrity and security. The literature database was actively maintained through regular updates, link verification, and periodic reviews to ensure completeness and accessibility. This systematic management facilitated efficient retrieval and utilization of literature for data analysis and research.

### 2.2 Establish of Data Collection Tables and Enumeration Values

A systematic meta‐like analysis approach was adopted to construct a comprehensive data collection framework for neurological diseases such as Alzheimer's disease (AD), Parkinson's disease (PD), and Huntington's disease (HD) [21]. Initially, a pre‐collection system was conducted to gather relevant information on conventional research methods, modeling techniques, and various indicators mentioned in the literature. Based on the pre‐analysis model, data collection forms were developed for each disease. These forms were structured into 11 sections to cover key aspects of neurological disease modeling and analysis: (1) Model Overview, (2) Strain Information, (3) Modeling Method, (4) Biologics, (5) Gene Editing, (6) Surgical Modeling, (7) Behavioral Changes/Specific Values, (8) Pathological Phenotypes, (9) Blood Biochemistry/Specific Values, (10) Cerebrospinal Fluid Biochemistry/Specific Values, and (11) Imaging. Sections (4–6) focused on detailed data collection related to distinct modeling types, while sections (7–11) encompassed behavioral (e.g., morris water maze, open field test), biochemical (e.g., neurotransmitter levels, protein expression), pathological (e.g., amyloid‐beta plaques, tau tangles), and imaging changes (e.g., MRI, PET scans) in animal models.

To ensure comprehensive coverage and minimize bias in literature selection, three investigators independently searched Google Scholar, PubMed, and Web of Science for articles published until April 2024. Moving forward, they summarized relevant information by reading reviews and supplemented missing or incomplete indicators discovered in the original data through research literature. For instance, model applications were categorized as “Phenotype and Symptom Comparison,” “Mechanistic Investigation,” “Behavior Studies,” “Pathological Study,” “Drug Screening,” “Gene Function Study,” and “Drug Efficacy Evaluation,” to enhance the consistency of data collection (Tables S1 and S2).

### 2.3 Data Curation and Integration

The Neurological Disease Animal Model Database (ND‐AMD) implemented standardized naming conventions to ensure consistency across key fields such as disease name, modeling type, genes, and species in model names. This comprehensive standardization extends to species and strain names. Within ND‐AMD, three primary categories of animal models are defined: induced animal models, genetically engineered animal models, and induced and genetically edited animal models.

Induced animal models are identified by the causal agent, strain, species, disease name, and model type. Examples include “6‐OHDA induced SD rat Parkinson's disease model” or “MPTP induced cynomolgus monkey Parkinson's disease model”. Conversely, genetically engineered animal models are designated by the altered gene, transgenic status, strain, species, disease, and model type. For instance, models may include “APP/PS1 transgenic C57BL/6J mouse Alzheimer's disease model”.

Furthermore, induced and genetically edited animal models encompass both induced and genetically modified elements, indicated by the causal agent, strain (including genetic modifications), species, disease name, and model type. After the collection of data, models and model applications are segregated within ND‐AMD, with post‐treatment animal models housed in the model application section and organized into respective categories.

To ensure data integrity and reproducibility, rigorous quality control measures were implemented, including manual verification of phenotype data and cross‐referencing with original studies. To streamline subsequent analyses, all phenotypic data and measurement units were standardized across all entries, ensuring consistency and comparability. This standardization process adhered to established protocols, allowing for seamless integration of behavioral, biochemical, and pathological parameters. The process of combining data involved meticulous collation and integration of heterogeneous datasets from various sources. Initially, raw data were retrieved from diverse repositories and research articles, each with its own format and structure.

Subsequently, data cleansing techniques were applied to rectify inconsistencies and errors, ensuring the integrity and accuracy of the compiled dataset. Utilizing SQL (Structured Query Language), data from disparate sources were harmonized and aggregated into a unified database schema [22]. This involved transforming data into a common format, standardizing variable names and units (e.g., using international units for biochemical measurements), and resolving discrepancies across datasets. SQL queries were employed to perform data transformation, manipulation, and aggregation, facilitating efficient data processing and organization.

### 2.4 Heterogeneity and Sensitivity Analysis

To ensure the consistency and robustness of the study results, both heterogeneity and sensitivity analyses were performed on the processed phenotypic data. Heterogeneity assessment was pivotal in determining the consistency across study results [23]. Each set of phenotypic data underwent rigorous testing to evaluate the null hypothesis, aiming to discern if primary study outcomes significantly differed. This evaluation encompassed both Cochran's *Q* test and the *I* ^2^ statistic. Cochran's *Q* test assesses if observed differences among study results are due to chance, but its accuracy may decrease with fewer studies. To supplement this, the *I* ^2^ statistic quantifies the proportion of total variation across studies attributable to heterogeneity. It ranges from 0% to 100%, with values indicating low (25%), moderate (50%), and high (75%) heterogeneity (*I* ^2^ = 100% × (*Q ‐* df)/*Q*). The utilization of Python's library named SciPy (version 1.11.1) and its stats models facilitated the computation of these heterogeneity metrics, ensuring the precision and accuracy of the analysis.

Sensitivity analysis was then executed to gauge the robustness of the overall findings [22]. This entailed systematically varying key parameters and re‐evaluating the outcomes to discern any notable changes. Sensitivity analysis operates on the principle of discerning how alterations in independent variables influence a particular dependent variable under a predetermined set of assumptions. In this context, the analysis involved excluding individual studies or subsets of data to observe their impact on the overall effect size and heterogeneity metrics. By employing Pandas (version 1.5.3) in Python, diverse scenarios were simulated through the modification of input parameters, followed by re‐execution of the analyses. Subsequently, the resultant outcomes were meticulously compared against the original findings to ascertain any discrepancies or variations, thereby affirming the stability and reliability of the conclusions drawn.

### 2.5 Comparative Phenotypic Analysis

The comparative analysis of phenotypic data involved meticulous classification and statistical examination based on sample sizes. For small sample sizes (*n* < 30), fine subdivision data such as clinical scores and weights were compared between groups using Welch's *t*‐test, chosen for its robustness against unequal variances and non‐normality. For medium sample sizes (30 ≤ *n* < 100), behavioral indicators were assessed through independent *t*‐tests or Analysis of Variance (ANOVA), depending on the data distribution, to accommodate varying group sizes and variance homogeneity. Python's SciPy library facilitated the computation of accurate *p*‐values for these tests. Additionally, the polyfit function in SciPy was utilized to fit a quadratic polynomial and describe data trends.

To enhance the rigor of the analysis, control variables were systematically managed using several approaches. Initially, stratified randomization was employed, dividing subjects into strata based on specific variables (e.g., a particular behavioral test or the type of experimental animal model) and then randomly assigning subjects within each stratum. Following stratification, group matching ensured similar distributions of specific variables across experimental groups without performing individual one‐to‐one matching.

During the statistical analysis, we controlled for variables using analysis of covariance (ANCOVA), which adjusts for the influence of control variables, allowing the assessment of the net effect of the main independent variable on the dependent variable. The ANOVA procedure, integrated with ANCOVA, was meticulously followed to compare means among groups. The process began with formulating null and alternative hypotheses: the null hypothesis (H0) posited no difference in means among the groups, while the alternative hypothesis (H1) suggested that at least one group mean was different. We computed the *F*‐statistic using sample data, incorporating the variation between group means relative to the variation within the groups. ANCOVA was applied to adjust for the influence of control variables by adding these variables to the model. This refinement allowed the *F*‐statistic to reflect the net effect of the primary independent variable after accounting for control variables.

*p*‐values were derived based on the adjusted *F*‐statistic, the number of groups, and the sample size, indicating whether the observed differences among group means were statistically significant. Decision‐making regarding the null hypothesis was guided by comparing the *p*‐value to a significance level (typically 0.05). A *p*‐value less than 0.05 led to the rejection of the null hypothesis, indicating significant differences in at least one group mean. Following a significant ANOVA result, post hoc analysis, such as Tukey's HSD test, was performed to identify specific group differences, further clarifying which groups differed from each other.

To ensure data quality and integrity, exploratory data analysis (EDA) was conducted using Python's NumPy (version 1.24.2) and Pandas libraries. This encompassed procedures such as handling missing values, duplicates, data type conversions, and data filtering. These processes were encapsulated into a method for comparative analysis. Visualization techniques, including dot plots, box plots, and scatter plots, were employed to enhance data interpretation using Python's matplotlib and Seaborn libraries. Furthermore, interactive plots were generated to facilitate a deeper understanding of the data trends. Reproducibility was ensured through the use of code repositories and version control for all analysis scripts.

### 2.6 Model Frequency Analysis

Comprehensive definitions and standardizations have been meticulously established for the disease categories, disease names, and research purposes associated with all animal models of neurological diseases within our database. The disease categories are classified into six major types, encompassing a total of 19 distinct disease names. To determine the frequency of application of these models, we conducted an extensive analysis of sample quantities derived from application data linked to relevant literature for each neurological disease animal model. Through this analysis, we identified the most frequently occurring application data, which allowed us to conduct a robust recommendation frequency analysis.

The results, showcasing the frequency of model usage within the database, are categorized based on the specific diseases and research purposes of interest to the users. This data is visualized using a pie‐donut chart, which provides a clear and intuitive representation of the distribution and prevalence of different animal models. The visualization tool used for this purpose is available at Highcharts v8.0 (https://www.highcharts.com/demo/highcharts/3d‐pie‐donut), which facilitates a deeper understanding of the trends and patterns in model usage.

### 2.7 Bibliometric Analysis

Text co‐occurrence network analysis was applied to analyze the terms in literature related to animal models of different neurological diseases. This analysis involved the identification of terms that frequently appear together within the literature corpus, indicating potential semantic or conceptual relationships.

We employed a weighted linking method, wherein the strength of the relationship between terms is determined by their co‐occurrence frequency. Specifically, VOSviewer software (version 1.6.14) was employed to construct co‐occurrence network diagrams [24]. This allowed for the visualization of associations among terms, with nodes representing terms and edges indicating the strength of their co‐occurrence relationship. To ensure the robustness of our analysis, a minimum occurrence threshold was set, requiring each term to appear at least twice within the corpus. Additionally, synonymous terms were manually merged, and irrelevant terms were excluded from the analysis to minimize redundancy and improve the accuracy of the data.

**FIGURE 1:**
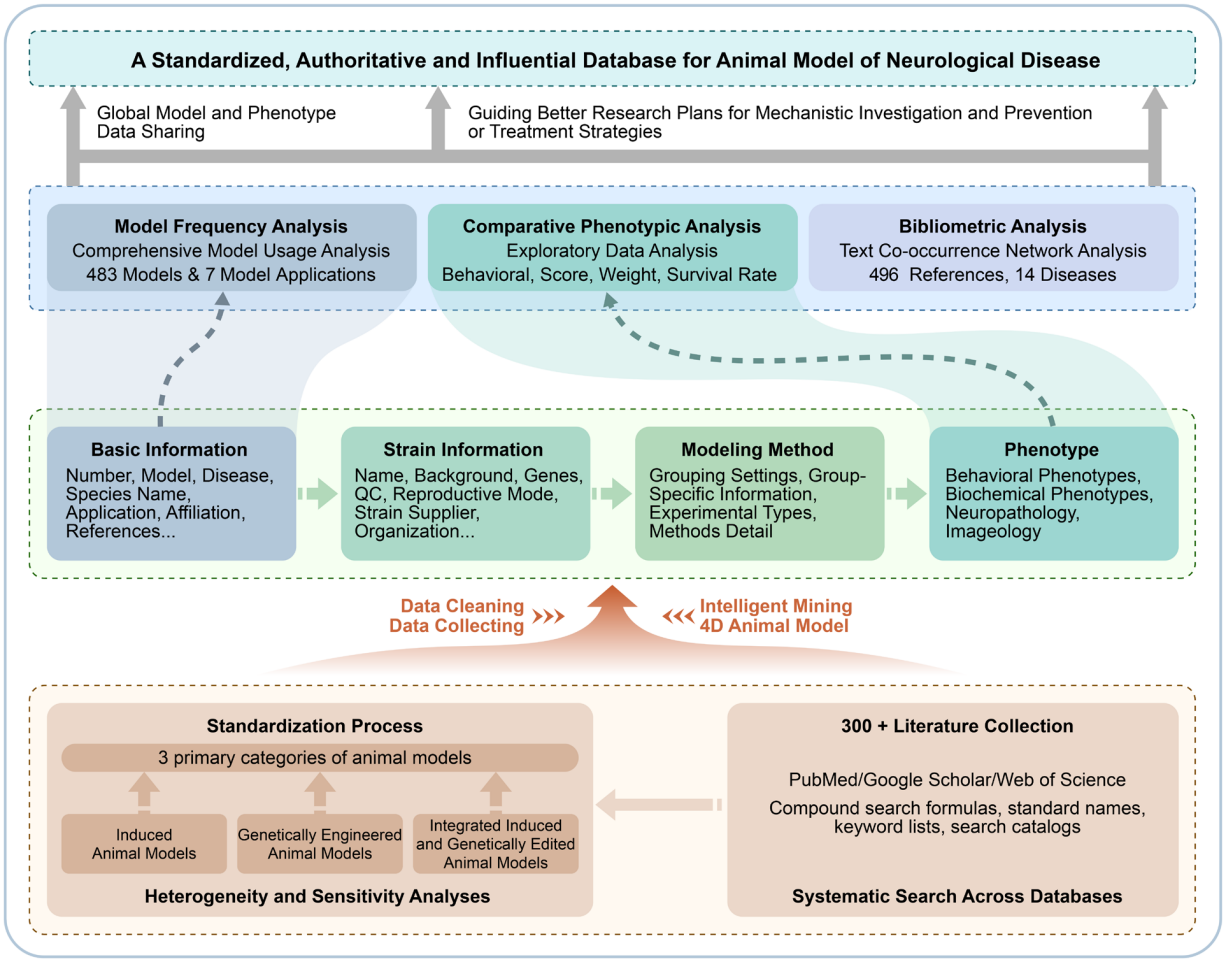
Overview of ND‐AMD framework and structure.

Furthermore, we utilized Python‐based Natural Language Processing (NLP) code to validate the precision and relevance of the word cluster outputs derived from the network analysis [25]. Specifically, we employed the NLTK (version 3.8.1) library, which offers a wide range of tools and algorithms for NLP tasks such as tokenization, stemming, and semantic analysis. This additional step will enable us to further refine our analysis and gain deeper insights into the relationships among terms in the literature corpus (Figure 1).

### 2.8 Web‐Interface Architecture and Development

The web interface was constructed using HTML5/CSS/JavaScript to establish the basic structure, style, and interactive functionalities. jQuery 1.3 was utilized as a supporting tool primarily for user interactions. Java 1.7 and Python 2.7 were employed to manage the website's business logic and handle data transmission. The backend system adopted the Enterprise Java Beans framework, with JBoss 6.0 serving as the middleware. Structured data was stored and managed using the relational database MySQL (version 5.7). Additionally, a static file server was built using Node.js (version 18.18), and Nginx was implemented for load balancing and reverse proxy. To ensure the security of data transmission, HTTPS and SSL/TLS protocols were employed. The system was designed with principles of openness, ease of operation, user‐friendly interface, reliability, and security, providing users with a unified and friendly operation, user‐friendly interface, reliability, and security. The web interface offered a bilingual version with options to switch between English and Chinese, catering to a wider audience. It was freely accessible to the public, promoting accessibility and inclusivity.

## 3 Result

### 3.1 Database Framework and Statistics

The ND‐AMD database comprises five primary sections: Home, Browse, Advanced Search, Analysis Tools, and Data Resources.

#### 3.1.1 Home Section

Home section contains various functional modules, including navigation by species for animal models of neurological diseases, categorized browsing, data statistics, data submission, data download, related news and literature, and citation information.

#### 3.1.2 The Database Content

**FIGURE 2:**
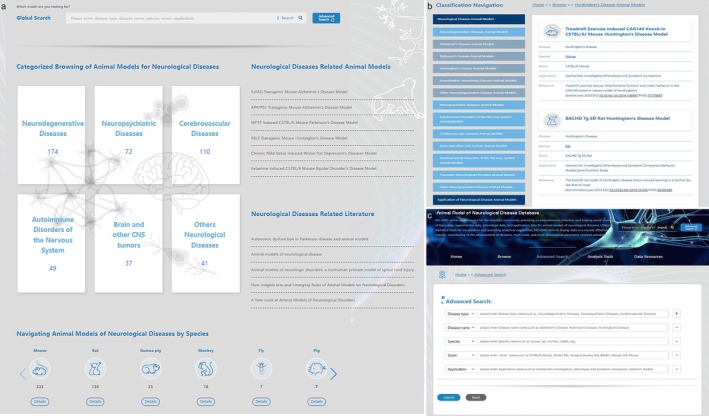
Model browsing and retrieval. (a) ND‐AMD database content; (b) Quickly browse neurological disease models by classification navigation; (c) Advanced search function with freely combined refined search queries.

ND‐AMD database includes a total of 483 animal models of neurological diseases, structured around a data‐model‐phenotype framework, covering eight major disease categories, containing neurodegenerative diseases (174 models), neuropsychiatric disorders (72 models), autoimmune disorders of the nervous system (49 models), cerebrovascular diseases (110 models), brain and other CNS tumors (37 models), developmental disorders of the nervous system (39 models), traumatic neurological disorders (2 models) and other neurological diseases, which encompass 21 additional subcategories, 152 strains (Figure 2a). The species are mainly mouse, rat, and monkey, with a total of 13 species. A series of genetically engineered animal models with different genes (knock‐in and knock‐out) were collected, mainly involving AD‐related genes (APP, PSEN) (Table 2 and Table S3).

**TABLE 2:** Database sample information.

Type	Content	Count
Species	Mouse, rat, monkey, rabbit, fly, pig, zebrafish, dog	13
Disease types	Neurodegenerative diseases, cerebrovascular diseases, neuropsychiatric diseases	8
Disease	Alzheimer's disease, Parkinson's disease, Huntington's disease, epilepsy, depression	21
Strains	C57BL/6 mouse, Wistar rat, Sprague Dawley rat, BALB/c mouse, ICR mouse	152
Genes	APP, PS1, ASIC1a, PINK1, SNCA, LRRK2, Bmal1	21
Models	APP/PS1 transgenic mouse Alzheimer's disease model, MPTP induced C57BL/6 mouse Parkinson's disease model, R6/2 transgenic mouse Huntington's disease model, chronic mild stress‐induced Wistar rat depression model	483

#### 3.1.3 Categorization and Browsing

ND‐AMD categorized animal models by the types of neurological diseases and species. The homepage provides quick access to major categories, redirecting users to the model browsing page. This page is further divided into models and model applications. Models are organized into eight categories of neurological disease animal models, displaying disease name, species, strain, model application, and references. This allows researchers to quickly browse relevant animal models (Figure 2b).

#### 3.1.4 Model Results Retrieval

On the advanced search function page, users have the options to select various parameters such as disease type (e.g., neurodegenerative diseases), disease name (e.g., Alzheimer's disease), species (e.g., mouse), strain (e.g., C57BL/6), and application (e.g., mechanistic investigation) to conduct advanced search. These parameters can be freely combined to refine search queries according to the user's requirements (Figure 2c). Additionally, users can utilize the global search function without restricting their search to specific parameters. By entering keywords such as disease type, disease name, species, strain, and application, users can quickly filter out corresponding animal models. ND‐AMD presents the search results in descending order of relevance to the search keywords, ensuring that the most relevant models are displayed prominently for user consideration.

#### 3.1.5 Model Data Presentation

The model details page is structured into four sections, each with corresponding navigation: basic information, strain information, modeling method, and phenotype.

#### 3.1.6 Basic Information Section

The section offers a summary of the model, including model number, strain number, reference number, model name, disease name, species name, modeling application, affiliation, and references. It serves to provide users with a quick overview of the model's fundamental characteristics.

#### 3.1.7 Strain Information Section

Here, users can find details about the strain name, containing strain name, background strain, genes involved, microbial quality control, reproductive mode, strain supplier, and organization. This section aims to provide comprehensive information about the genetic background and source of the model.

#### 3.1.8 Modeling Method Section

This section outlines the experimental settings and methods used in the model, including grouping settings, group‐specific information, experimental types, and detailed experimental methods. It offers insights into the experimental design and methodology employed in generating the model data.

#### 3.1.9 Phenotype Section

Phenotypic data are categorized into behavioral phenotypes, biochemical phenotypes, neuropathology, and imageology. Biochemical phenotypes are further subdivided into blood biochemistry and cerebrospinal fluid biochemistry, highlighting changes in phenotypic indicators between the modeling and control groups. Phenotype information is presented interactively through graphical formats such as line charts, bar charts, and box plots, allowing users to visualize specific values for different groups in real time. Users can export the phenotype data in various formats (e.g., png, jpeg, pdf, svg) for further analysis. Additionally, brief descriptions of relevant clinical and neuropathological characteristics, based on corresponding literature, are provided to enhance understanding.

Furthermore, we have developed a drug screening database (https://www.uc‐med.net/DrugScreen/) based on animal models, which includes 81 drug screening experimental datasets for degenerative diseases such as AD, PD, HD, etc. This database serves as a valuable resource for drug discovery research [26].

### 3.2 Comparative Phenotypic Analysis

**FIGURE 3:**
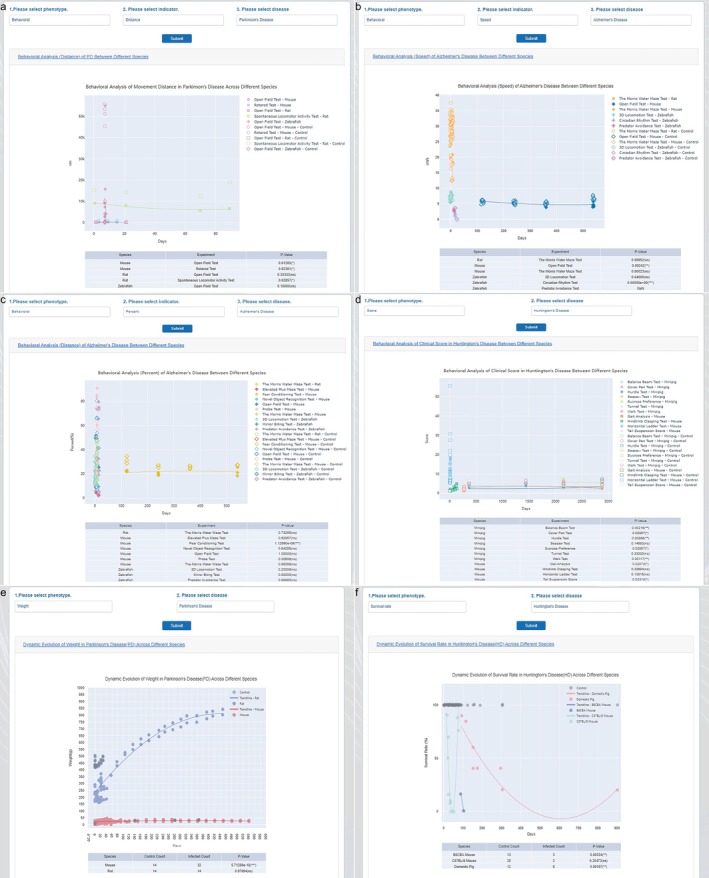
Comparative Phenotypic Analysis across Datasets Using Integrated 3D Phenotypic Data and 4D Temporal Information. (a–d) Comparative behavioral analysis of different neurological diseases across species: (a) Movement distance in PD; (b) Movement speed in AD; (c) Movement percentage in AD; (d) Clinical scores in HD; (e,f) Dynamic evolution of key metrics in neurological diseases across species: (e) Weight in PD; (f) Survival rate in HD.

The comparative phenotypic analysis implemented variations of different phenotype indicators across various species and displayed them interactively. This approach achieved multi‐angle, multi‐level phenotypic analysis, addressing the challenges of cross‐species comparisons due to inter‐species differences. Users could first select a phenotype (such as behavior, score, weight, survival rate), then choose a specific indicator, and finally select a particular neurological disease. After submission, the phenotype analysis charts and corresponding data tables (including *p*‐values) were displayed interactively (Figure 3a–f). Through cross‐datasets analysis, we could gain insights into complex neurological diseases such as AD. By integrating three‐dimensional (3D) phenotypic data and four‐dimensional (time) information of diseases, a comprehensive dynamic description of disease progression, including early symptoms, mid‐term progression, and late‐stage manifestations, could be obtained. Phenotypic data of animal models at different time points revealed the progression of the disease [27]. This analysis serves as a branch of the comparative medicine big‐data platform, providing phenotypic analysis support.

For example, the integrated analysis of behavioral studies involved eight indicators: Distance, Percent, Speed, Time, Frequency, Angular Velocity, Degree Inclined, and Grip Strength. These indicators investigated behavioral changes from different perspectives and levels (basic behavioral patterns, neural system complexity, impact of diseases or injuries) (Figure 3a–c). Distance and Speed measured the range and velocity of animal movement; Time and Frequency described the duration and frequency of behavioral occurrences, while Angular Velocity and Degree Inclined focused on the direction and inclination of behavior. The integrated analysis of ethology could indirectly explore the complexity and functions of the neural system. The results demonstrated that, compared to other species, neurodegenerative diseases had a more pronounced impact on the motor ability of mice. For instance, in PD models, a significant reduction in movement distance and speed was observed, reflecting hallmark symptoms such as bradykinesia and impaired locomotor coordination [28, 29]. Similarly, AD models exhibited deficits in grip strength and spatial navigation, consistent with progressive neuromuscular decline and cognitive impairment. These findings highlight the varying degrees of motor dysfunction across different neurological conditions and emphasize the sensitivity of specific behavioral indicators in disease modeling.

Integrating behavioral analysis such as the morris water maze test, open field test, 3D locomotion test, circadian rhythm test, and predator avoidance test held significant importance, specifically in comprehensively understanding animal behavioral characteristics such as cognitive abilities, emotion, autonomous activity, and motor abilities. This integration revealed the relationship between physiological rhythms and behavior (circadian rhythms) and assessed laboratory animals' survival instincts and adaptability (predator avoidance). By combining the results of these tests, a more comprehensive assessment of the impact of neurological diseases or injuries on animal behavior at different times could be made. Different testing methods provided information about animal behavior from various angles, and integrating these data complemented and validated each other, improving research accuracy and reliability.

### 3.3 Model Frequency Analysis

Model frequency analysis provides users with the most commonly used models based on their research needs, along with detailed information and references. Depending on the diseases and research purposes associated with the models, statistical analyses are conducted to guide users in selecting the appropriate models. Users can learn about the most frequently used models, evaluate the citations and phenotypes of each modeling method, and utilize this information to guide their experiments.

Users are guided through a step‐by‐step process to refine their search, by sequentially selecting the disease type, specific disease name and research purposes (phenotype and symptom comparison, mechanistic investigation, gene function study, behavior studies, pathological study, drug efficacy evaluation, drug screening, and drug evaluation research). After submitting their choices, the model frequency results are displayed based on “model applications” and “frequency of model occurrences”. A list of models, including model names, numbers and reference, were displayed. For instance, when users selected “Neurodegenerative Diseases”, the disease options were changed accordingly to include neurodegenerative diseases available in ND‐AMD (AD, PD, HD and etc.). Users could then choose a disease of interest (e.g., “Alzheimer's disease”) and selected one or multiple research purposes (e.g., single‐selected “Mechanistic investigation”). A list of the most used Alzheimer's disease models for mechanistic studies would be recommended. Clicking on a model number could jumped to a detailed page for the corresponding animal model of neurological disease. The high frequency of models appearance indicated its frequent used by researchers, providing users with guidance for model selection in their subsequent experimental design.

**FIGURE 4:**
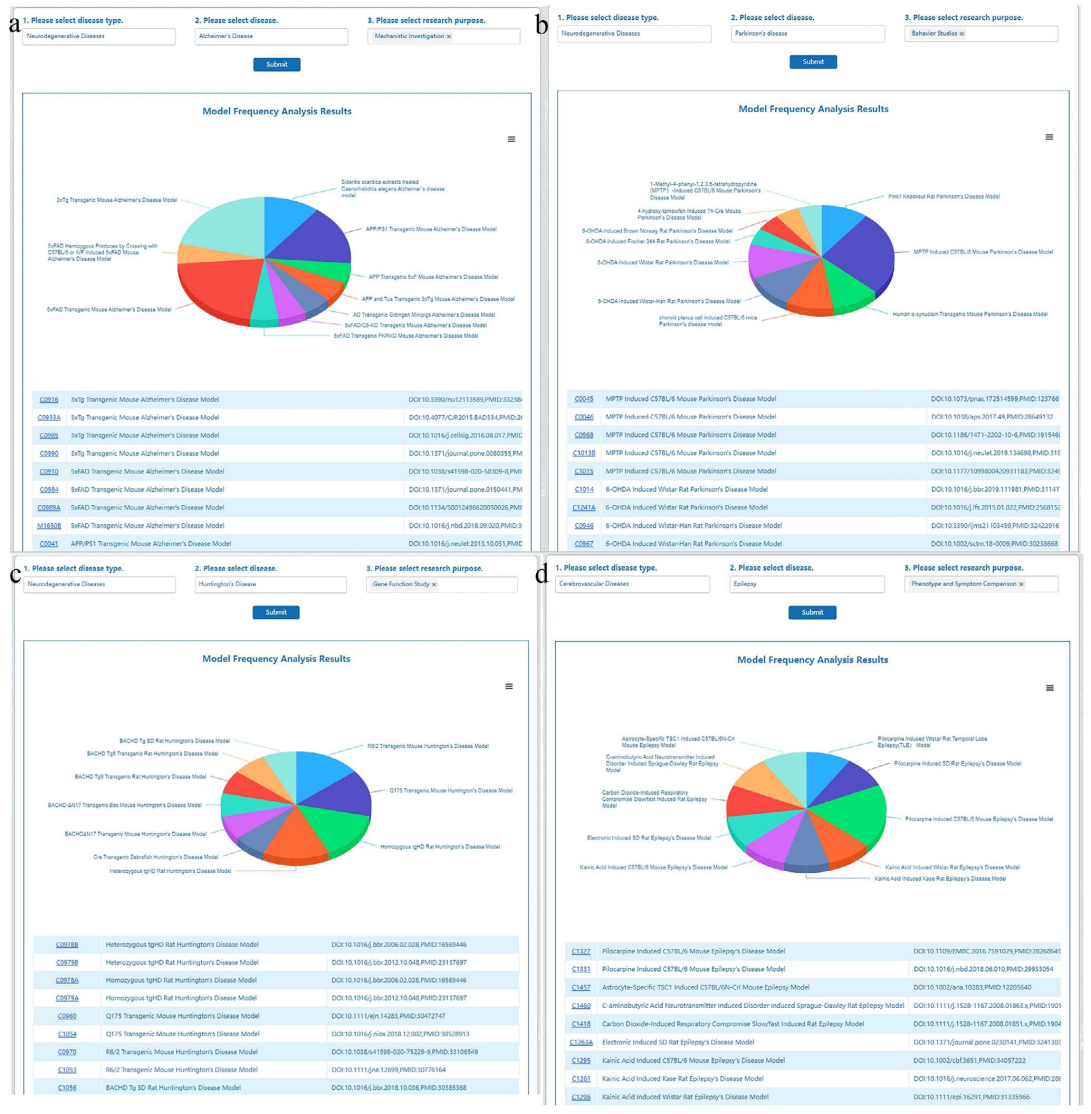
Model frequency analysis via Pie‐Donut chart. (a) Frequency distribution of the most commonly used models in AD mechanistic research. (b) Frequency distribution of the most commonly used models in PD behavioral studies. (c) Frequency distribution of the most commonly used models in HD gene function research. (d) Frequency distribution of the most commonly used models for phenotype and symptom comparison in epilepsy research.

The analysis revealed that the 3xTg transgenic mouse model was the most commonly used model in AD mechanism research (Figure 4a). This model harbors mutations in APP, PSEN1, and MAPT, enabling it to recapitulate key pathological features of AD, including amyloid plaque formation, neurofibrillary tangles, and progressive cognitive decline. Notably, 3xTg mice exhibit impaired spatial learning and memory, closely mirroring the cognitive deficits observed in human AD patients [30, 31, 32]. In PD behavioral studies, the neurotoxin‐induced model emerged as the most widely used (Figure 4b). This model employs neurotoxic substances such as 6‐hydroxydopamine (6‐OHDA) and 1‐methyl‐4‐phenyl‐1,2,3,6‐tetrahydropyridine (MPTP) to selectively destroy dopaminergic neurons in the substantia nigra‐striatum pathway, mimicking the motor and non‐motor symptoms of PD. The 6‐OHDA and MPTP models are particularly valuable for their ability to replicate the progressive loss of dopaminergic neurons and the resulting bradykinesia, rigidity, and tremors, which are hallmark motor deficits in human PD [33, 34, 35, 36]. For HD gene function research, the R6/2 transgenic mouse and heterozygous tgHD rat models were identified as the most frequently utilized. These models carry CAG repeats in the HTT gene, leading to the expression of mutant huntingtin protein, which drives disease pathology. Both models exhibit progressive motor dysfunction, cognitive decline, and neuropathological hallmarks such as striatal neuron loss and inclusion body formation, closely resembling the human HD phenotype (Figure 4c) [37, 38, 39, 40]. In epilepsy research, particularly for phenotype and symptom comparison, the pilocarpine‐induced model was the most commonly employed (Figure 4d). This model induces status epilepticus, followed by the development of spontaneous recurrent seizures, hippocampal sclerosis, and behavioral changes, effectively replicating the pathological and clinical features of human temporal lobe epilepsy [41, 42, 43].

### 3.4 Bibliometric Analysis

By utilizing knowledge graphs, extracting and organizing relevant terminology, we mined the research focuses of different neurological diseases, investigated major research trends, and provided references and insights for researchers. This analysis covered: Alzheimer's Disease (51 articles), Parkinson's Disease (51 articles), Huntington's Disease (45 articles), Bipolar Disorder (32 articles), Depression (38 articles), Schizophrenia (18 articles), Epilepsy (39 articles), Stroke (30 articles), Guillain‐Barré Syndrome (20 articles), Multiple Sclerosis (18 articles), Meningioma (41 articles), CNS Tumor (16 articles), Attention Deficit Hyperactivity Disorder/Autism Spectrum Disorder (80 articles) and Myasthenia gravis (17 articles). When users selected disease types, related diseases, and submitted their query, the database displays the frequency of related terms. Higher term frequencies indicate more frequent research.

**FIGURE 5:**
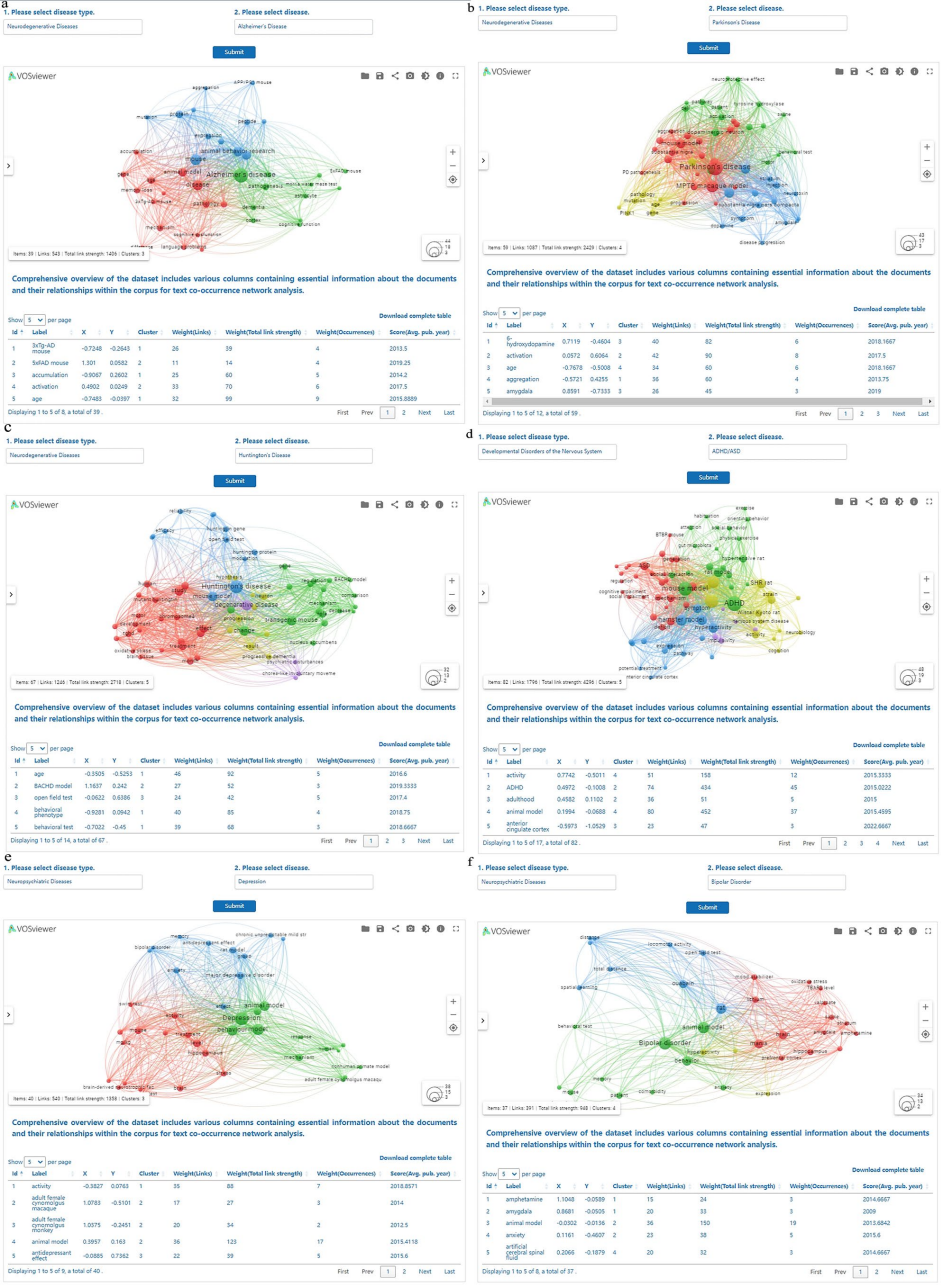
Bibliometric analysis of neurological disease research using co‐occurrence Network Diagrams. (a) Co‐occurrence network analysis of literature on AD. (b) Co‐occurrence network analysis of literature on PD. (c) Co‐occurrence network analysis of literature on HD. (d) Co‐occurrence network analysis of literature on ADHD and ASD. (e) Co‐occurrence network analysis of literature on depression. (f) Co‐occurrence network analysis of literature on BD.

For example, research on AD focused on the abnormal aggregation of tau protein and amyloid‐beta (Aβ), both of which are central to the disease's pathogenesis. These pathological hallmarks are accompanied by neuroinflammation, neurodegeneration, oxidative stress, and synaptic dysfunction, all contributing to progressive cognitive decline and memory impairment. Genetically engineered animal models are widely used to study these mechanisms, with cognitive deficits often evaluated through the morris water maze test, which assesses spatial learning and memory (Figure 5a) [44, 45, 46]. In PD research, text co‐occurrence network analysis showed that neurotoxin‐induced models and gene‐editing models are the predominant approaches for establishing PD animal models. These studies examined changes in neurotransmitter levels, such as dopamine and acetylcholine, and explored the deposition of α‐synuclein aggregation and the effects of genetic mutations. The extent of dopamine neuron loss and its distribution in different brain regions were also examined. Behavioral tests, such as gait analysis and beam walking tests, are commonly used to evaluate motor deficits and assess the validity of these models in mimicking clinical PD symptoms. Among various PD models, the MPTP‐induced monkey model is widely regarded as the “gold standard”, owing to its strong reproducibility of PD symptoms and its anatomical and functional resemblance to the human nervous system. This model has been instrumental in validating clinical treatment strategies (Figure 5b) [47, 48, 49]. HD animal model literature focused on pathological and physiological changes such as neuronal loss, synaptic dysfunction, and neurofibrillary pathology. A key research focus is the progression of neuronal degeneration in the striatum and cortex, the regions most severely affected by HD. Additionally, studies have investigated gene expression changes associated with HTT mutations, along with alterations in synaptic protein levels and neurotransmitter systems, particularly dopamine. Advanced neuroimaging techniques, such as MRI, have been used to assess brain structural changes, including volume loss and white matter degeneration (Figure 5c) [50]. Research on ADHD/ASD animal models focused on pathophysiological mechanisms, including neurotransmitter imbalances, disrupted neural circuits, and altered gene expression patterns. Social impairment assessments often involved the three‐chamber social test, investigating the role of genetic factors in pathogenesis, and considering the influence of environmental factors on disease development, such as the impact of maternal environment on fetal neural development. Neuroimaging techniques have provided critical insights into structural and functional changes in the brain, helping to unravel the neural basis of these disorders (Figure 5d) [51, 52]. In depression research, animal models commonly exhibit depressive‐like behaviors, such as reduced locomotion, social withdrawal, and impairments in learning and memory. Many studies have focused on alterations in neurotransmitter systems, particularly serotonin and norepinephrine, and their impact on mood regulation and cognitive function. Furthermore, structural and functional changes in key brain regions involved in emotional processing, such as the amygdala and hippocampus, have been extensively investigated. Behavioral tests, including the forced swim test and tail suspension test, are widely used to assess depressive‐like phenotypes in rodent models (Figure 5e) [53, 54]. Research on animal models of BD, focused on abnormal emotional behaviors such as agitation, depression, and emotional instability. These studies explored changes in neurotransmitter changed in neurotransmitter systems, including dopamine, glutamate, and serotonin, as well as alterations in brain circuits involved in mood regulation (Figure 5f) [55].

## 4 Discussion

The concept of “animal welfare” is widely defined by five freedoms: freedom from hunger and thirst, freedom from discomfort, freedom from pain, injury or disease, freedom from fear and distress, and the freedom to express normal behavior. However, ensuring all five freedoms for laboratory animals remains challenging, as experimental procedures often require necessitate controlled physiological or pathological manipulations. The widely accepted ethical framework balancing scientific research and animal suffering is the *3Rs principle*: Replacement, Reduction and Refinement [56, 57].

The development of ND‐AMD aligns with this principle by enabling the efficient utilization of existing data, thereby minimizing the reliance on live animal experiments. By providing researchers with comprehensive datasets on animal models of neurological diseases, the database reduces redundant data collection and facilitates more targeted experimental designs. For instance, researchers can identify the most suitable animal models for specific research objectives, such as drug screening or mechanistic investigations, thereby accelerating the identification of novel therapeutic targets.

Analysis of ND‐AMD data reveals notable trends in the use of laboratory animals. Small animals (e.g., mice, rats, zebrafish, *Drosophila melanogaster* , *Caenorhabditis elegans* ) employed in epilepsy and cerebral injury studies were typically aged 2–4 months, while animals under 1 month of age were predominantly used in developmental studies involving gene editing. In contrast, animals older than 3 months were employed for chronic diseases and neurodegenerative diseases. Large animal models (e.g., rhesus macaques, cynomolgus macaques, dogs) were typically between 6 and 9 years old to simulate aging‐related conditions, particularly for AD and PD research (see Table S4).

Behavioral experiments recorded in ND‐AMD predominantly focus on motor function, limb‐specific activity, exploratory behavior, and adaptability to novel environments. Key areas of interest include the evaluation of neural system development, cognitive impairments, and motor coordination. The most prevalent purposes include anxiety‐like behaviors, motor function, and locomotor exploratory behavior, reflecting the crucial role of these aspects in neurological research. Anxiety‐like behaviors, accounting for 15.83%, highlight the importance of understanding stress responses. Motor function studies, at 12.92%, are essential for evaluating movement disorders, while locomotor exploratory behavior, representing 8.99%, is critical for assessing overall activity and interaction with new stimuli (see Table S5). The efficient use of existing data shortens the research cycle, improves research efficiency, and promotes the development of alternatives to laboratory animals. Through the collection, processing, and analysis of disease animal models and related comparative medical data, scientific research is promoted, achieving a virtuous cycle between data and scientific research. This process realizes the integration and informatization of scattered research resources and promotes the transformation of research methods from merely recording the characteristics of animal models to software‐based feature recognition and prediction centered on multimedia computers. Consequently, a multi‐species, multi‐model, multi‐factorial disease animal model system is formed.

Despite the indispensable role of animal models in neurological research, ensuring their ethical and practical application remains crucial. These models not only replicate key pathological features of neurological disorders but also bridge the gap between fundamental research and clinical applications. Compliance with *FAIR* data principles (Findable, Accessible, Interoperable, and Reusable) and the *TRUST* principles for digital repositories (Transparency, Responsibility, User focus, Sustainability, and Technology) [58, 59] ensures the reliability and accessibility of the database. Such databases can help identify common mechanisms or targets across different diseases, leading to the development of more effective treatments.

To maintain ND‐AMD remains comprehensive and up to date, a “Data Submission” function enables researchers to contribute new datasets. Users can download standardized data collection forms, submit completed model descriptions, and undergo a verification process before formal data integration. Periodic literature reviews will be conducted to incorporate newly published findings, ensuring the database evolves in tandem with advances in the field. Additionally, a “Feedback Mechanism” allows users to report issues and suggest improvements, ensuring that the database continues to meet the needs of the scientific community. Future developments will focus on integrating emerging data types, such as single‐cell sequencing and multi‐omics datasets, and implementing advanced analytical tools, including machine learning‐based predictive models. Expanding international collaborations will further enhance coverage across diverse species and disease models, reinforcing ND‐AMD's role as a global hub for neurological disease research.

Previous comparative analysis studies often focused on the exploration of genetic information in biological genomes, conducting analyses on gene encoding sequences, structures, homology, as well as comparisons at the genomic and transcriptomic levels. ND‐AMD serves as a resource for the scientific community, facilitating collaboration and knowledge sharing. Researchers can access detailed information about various animal models, including characteristics, experimental protocols, and visualized outcomes, ultimately optimizing experimental design and accelerating discoveries in neurological research.

## 5 Conclusion

A centralized database, such as ND‐AMD, facilitates large‐scale comparative analyses across various animal models, strains, disease types, and experimental conditions. This comprehensive integration enables researchers to identify patterns, trends, and potential therapeutic targets that may not be apparent from isolated studies. By aggregating and standardizing data, the database supports robust meta‐analyses and cross‐study comparisons, enhancing reproducibility and reliability. Furthermore, ND‐AMD promotes the efficient utilization of existing datasets, minimizing redundant experimentation and aligning with ethical principles in animal research. The centralized database serves as a critical resource for advancing our understanding of neurological diseases and accelerating the development of effective treatments. As ND‐AMD continues to evolve by integrating advanced methodologies and expanding its scope, it has the potential to become a valuable resource for the global neuroscience research community.

## Author Contributions

**Yue Wu:** designed the study, conceptualized the analysis methodology, wrote the main manuscript text, and substantively revised it. **Lu Li:** responsible for acquisition, analysis, interpretation of data, software development, and manuscript revision. **Yi‐Tong Li:** participated in data curation. **Lei Zhang:** provided validation and visualization. **Shuang Gong:** responsible for software development. **Yang Zhang:** oversaw data visualization, validation, and supervision. **Jue Wang:** engaged in acquisition and interpretation of data. **Ling Zhang:** involved in data acquisition. **Qi Kong:** led the conceptualization, methodology, funding acquisition, and manuscript review and editing. All authors reviewed the manuscript.

## Ethics Statement

The authors have nothing to report.

## Consent

The authors have nothing to report.

## Conflicts of Interest

The authors declare no conflicts of interest.

## Supporting information


**Table S1.** Model outline acquisition sheet.
**Table S2.** Data collection and description table.
**Table S3.** Detailed sample information of animal model of neurological disease.
**Table S4.** Age distribution of laboratory animals in ND‐AMD.
**Table S5.** Distribution of behavioral experiment purposes in ND‐AMD database for animal models.

## Data Availability

All data supporting the findings of this study are available within the paper and its Supplementary Information and have been deposited and download in ND‐AMD database (accessible at https://www.uc‐med.net/NDAMD). Supplementary data related to this article are available online.
