# Supplementary material for: ND‐AMD: A Web‐Based Database for Animal Models of Neurological Disease With Analysis Tools
Source: CNS Neurosci Ther. 2025 May 9;31(5):e70411. doi: 10.1111/cns.70411 (PMC12063205; doi:10.1111/cns.70411)
Supplement: Supplementary file 1 — Table S1. Model outline acquisition sheet. Table S2. Data collection and description table. Table S3. Detailed sample information of animal model of neurological disease. Table S4. Age distribution of laboratory animals in ND‐AMD. Table S5. Distribution of behavioral experiment purposes in ND‐AMD database for animal models. [file CNS-31-e70411-s001.zip › cns70411-sup-0005-TableS2.docx]

1. Table 1 Model Summary

| 1.1 | ID | Resource number and serial number generated by platform. |
| --- | --- | --- |
| 1.2 | Model Number | Unique identifier number assigned to each model. |
| 1.3 | Strain Number | Assigned as S + four-digit number (e.g., S0001); a single literature source may reference multiple strains. |
| 1.4 | Literature Number | L - Serial number of assigned to literature resources collected by the platform. |
| 1.5 | Modeling Application | Categorized into Phenotype and Symptom Comparison, Mechanistic Investigation, Behavior Studies, Pathological Study, Drug Screening, Gene Function Study, and Drug Efficacy Evaluation. |
| 1.6 | Model Name (EH) | Includes disease name, modeling type, genes, species, and strain. |
| 1.7 | Model Name (CN) | Follows the same naming order as the Chinese version. |
| 1.8 | Strain | Standardized strain name used for modeling. |
| 1.9 | Specie | Taxonomic species of the modeled organism. |
| 1.10 | Model Classification | Categorized as either scientific research models or business models. |
| 1.11 | Accession Number | Same serial number as the literature number. |
| 1.12 | Research and Development Unit | Name and wbsite links of institutions involved in model development. |

1. Table 2 Modeling Strain

| 2.1 | Strain Number | Matches the ID assigned to each strain. |
| --- | --- | --- |
| 2.2 | Chinese Name | Standardized Chinese nomenclature for the strain. |
| 2.3 | English name | Internationally recognized strain name. |
| 2.4 | Latin Name | Scientific Latin name of the species. |
| 2.5-2.13 | Taxonomic Classification | Includes Kingdom, Phylum, Class, Order, Family, Genus (italicized, first letter capitalized), Species, and Subspecies. |
| 2.14 | Strain Name | Genetically classified and standardized nomenclature. |
| 2.15 | Background Strain | Genetic background of nsuring consistency and reproducibility in experiments. |
| 2.16 | Genes Involved | Key genes associated with the strain. |
| 2.17 | Strain Supplier | Institution and country where the strain was preserved before introduction. |
| 2.18 | Related Substrain | Substrain derived from the original strain. |
| 2.19 | Cultivation Year | Year when the variety and strains were successfully cultivated. |
| 2.20 | Number of Generations (min) | Number of reproductive generations, including pre- and post-introduction into the resource library. If unknown, marked as '?'. The time of the number of generations is indicated in parentheses after the number of generations. |
| 2.21 | Microbial Quality Control Level | Classified as conventional (CV), clean (CL), specific pathogen free (SPF), germ free (GF), and gnotobiotic (GN). |
| 2.22 | Reproduction status | Description of reproductive patterns for the strain. |
| 2.23 | Breeding Method | Techniques employed to maintain strain genetic integrity. |

1. Table 3 Biological Agents Modeling

| 3.1 | Group Number | Subdivide the groups under the model number. |
| --- | --- | --- |
| 3.2 | Modeling/Control | Distinguishes between modeling and control groups. |
| 3.3-3.4 | Group Name (CH/EN) | Naming follows the format: modeling method + species + inducing factors. |
| 3.5-3.6 | Specific Method & Injection Location | Detailed procedural information for modeling, including precise injection locations. |
| 3.7-3.8 | Specific Injection Area & Inducer Name | Specifies target site and substance used in the model. |
| 3.9-3.10 | Modeling Dose (MIN/MAX) | Defines the minimum and maximum injection doses administered. |
| 3.11-3.14 | Modeling Gender & Group Size | Specifies sex distribution of experimental animals, including male (m), female (f), and total (a) group sizes. |
| 3.15-3.22 | Modeling Parameters | Includes factor dose, age, weight, and duration of modeling (MIN/MAX). |
| 3.23-3.24 | Culture & Environmental Conditions | Defines humidity and diurnal cycle. |
| 3.25 | Note | Additional relevant information. |

1. Table 4 Gene Editing Modeling

| 4.1 | Group Number | Unique identifier for each experimental group within the study. |
| --- | --- | --- |
| 4.2 | Modeling/Control Classification | Specifies whether the group serves as a modeling (experimental) group or a control group. |
| 4.3 | Group Name | Standardized nomenclature incorporating the modeling technique, animal species, and key modeling factors. |
| 4.4 | Detailed Modeling Methodology | Comprehensive description of the applied gene-editing technique and experimental procedure. |
| 4.5 | Targeted Mutant Genes | List of mutated genes introduced in the modeling process. |
| 4.6 | Gene Reference Links | External links to gene databases providing detailed genomic information. |
| 4.7 | Laboratory Animal Sex | Specifies the sex of the animals used: male, female, or mixed. |
| 4.8 | Sample Size (Male) | Number of male animals included in the modeling experiment. |
| 4.9 | Sample Size (Female) | Number of female modeling included in the modeling experiment. |
| 4.10 | Sample Size (Total) | Total number of all modeling animals participating in the experiment (if sex-specific data are unavailable). |
| 4.11 | Modeling Age (MIN) | Minimum age of modeling animals participating in the experiment. |
| 4.12 | Modeling Age (MAX) | Maximum age of modeling animals participating in the experiment. |
| 4.13 | Modeling Weight (MIN) | Minimum body weight of modeling animals participating in the experiment. |
| 4.14 | Modeling Weight (MAX) | Maximum body weight of modeling animals participating in the experiment. |
| 4.15 | Minimum Duration of Modeling (Days) | Shortest duration (in days) required to establish the disease model. |
| 4.16 | Maximum Duration of Modeling (Days) | Longest duration (in days) required to establish the disease model. |
| 4.17 | Housing and Environmental Conditions | Description of the environmental conditions, including ventilation, bedding, and enrichment. |
| 4.18 | Minimum Humidity (%) | Lowest recorded humidity level in the animal facility. |
| 4.19 | Maximun Humidity (%) | Highest recorded humidity level in the animal facility |
| 4.20 | Humidity Variation Across Phases | Changes in humidity levels corresponding to different experimental phases. |
| 4.21 | Minimum Temperature (°C) | Lowest recorded environmental temperature during the study. |
| 4.22 | Maximum Temperature (°C) | Highest recorded environmental temperature during the study. |
| 4.23 | Temperature Variation Across Phases | Changes in temperature corresponding to different experimental phases. |
| 4.24 | Light-Dark Cycle (h) | Duration of light and dark periods per 24-hour cycle. |
| 4.25 | Gene Editing Approach | Specific gene-editing method employed, such as CRISPR-Cas9, TALEN, or ZFN. |
| 4.26 | Additional Note | Any supplementary details relevant to the modeling process. |

1. Table 5 Surgical Approach-Based Animal Modeling

| 5.1 | Group Number | Subdivide the groups under the model number. |
| --- | --- | --- |
| 5.2 | Modeling/Control Classification | Indicates whether the group serves as an experimental (modeling) group or a control group. |
| 5.3 | Group Name (Chinese) | Standardized group designation in Chinese, incorporating the surgical modeling method, animal species, and key experimental factors. |
| 5.4 | Group Name (English) | Standardized group designation in English, following the same format as the Chinese nomenclature. |
| 5.5 | Surgical Procedure | Surgical technique used for for disease modeling. |
| 5.6 | Surgical Site | Anatomical location of the surgical intervention. |
| 5.7 | Administered Drug | Name of the drug administered to the animals during the surgical procedure. |
| 5.8 | Drug Concentration | Concentration of administered drug. |
| 5.9 | Drug Dosage | Total dosage of the administered drug. |
| 5.10 | Injection Site | IAnatomical location of drug administration. |
| 5.11 | Age at Injection (Weeks) | Age (in weeks) of the animals at the time of drug administration. |
| 5.12 | Experimental Animal Sex | Specifies the sex of the animals used: male, female, or mixed. |
| 5.13 | Sample Size (Male) | Number of male modeling animals participating in the experiment. |
| 5.14 | Sample Size (Female) | Number of female modeling animals participating in the experiment. |
| 5.15 | Sample Size (Total) | The number of all modeling animals participating in the experiment (if sex-specific data are unavailable). |
| 5.16 | Minimum Age (Weeks) | Youngest age (in weeks) of animals at the initiation of the modeling experiment. |
| 5.17 | Maximum Age (Weeks) | Oldest age (in weeks) of animals at the initiation of the modeling experiment. |
| 5.18 | Minimum Body Weight (g) | Lowest recorded body weight (in grams) of animals before modeling. |
| 5.19 | Maximum Body Weight (g) | Highest recorded body weight (in grams) of animals before modeling. |
| 5.20 | Minimum Duration of Modeling (Days) | Shortest duration (in days) required to establish the disease model. |
| 5.21 | Maximum Duration of Modeling (Days) | Longest duration (in days) required to establish the disease model. |
| 5.22 | Housing and Environmental Conditions | Description of the environmental conditions, including ventilation, bedding, and enrichment. |
| 5.23 | Minimum Humidity (%) | Lowest recorded humidity level in the animal facility. |
| 5.24 | Maximum Humidity (%) | Highest recorded humidity level in the animal facility. |
| 5.25 | Humidity Variation Across Phases | Changes in humidity levels corresponding to different experimental phases. |
| 5.26 | Minimum Temperature (°C) | Lowest recorded environmental temperature during the study. |
| 5.27 | Maximum Temperature (°C) | Highest recorded environmental temperature during the study. |
| 5.28 | Temperature Variation Across Phases | Phase temperature for modeling animals. |
| 5.29 | Light-Dark Cycle | Specification of the diurnal cycle (e.g., 12-hour light/12-hour dark). |
| 5.30 | Modeling Technique | Detailed description of the surgical modeling methodology. |
| 5.31 | Additional Note | Any other relevant details, including deviations, special conditions, or references to literature. |

1. Table 6 Behavioral Assessments

| 6.1 | Observation Count | Number of phenotypic observations recorded. |
| --- | --- | --- |
| 6.2 | Mean Age | Average age of the animals at the time of behavioral testing. |
| 6.3 | Behavioral Test | Detailed protocol of the behavioral test conducted, including paradigms such as the Morris water maze, radial arm maze, multiple T-maze, and open field test. |
| 6.4 | Experimental Purpose | Purpose of the behavioral assessment, which varies based on the disease model (e.g., evaluation of spatial learning and memory, associative learning, motor function, or cognitive function). |
| 6.5 | Behavioral Indicator | Specific metrics assessed, including cognitive function indicators, motor function indicators, anxiety- and depression-related indicators, social behavior indicators, sensory function indicators, and reward/motivation indicators. |
| 6.6 | Test Date | Date on which the behavioral assessment. |
| 6.7 | Measured Value | Recorded outcome of the behavioral assessment. |
| 6.8 | Unit of Measurement | Standardized international units for quantitative assessment. |
| 6.9 | Additional Note | Additional relevant information. |

1. Table 7 Pathologic Phenotypes

| 7.1 | Observation Count | Number of phenotypic observations recorded. |
| --- | --- | --- |
| 7.2 | Pathological Assessment Protocol | Systematic framework for evaluating structural, molecular, and functional abnormalities associated with disease states. |
| 7.3 | Pathological Manifestations | Detailed characterization of pathological alterations, including structural, molecular, and functional abnormalities. |
| 7.4 | Pathological Indicator | Specific indicators used for assessment, such as structural indicators, molecular markers, functional metrics, and temporal changes. |
| 7.5 | Testing Days | Date on which the pathological evaluation was performed. |
| 7.6 | Measured Value | Quantitative or qualitative outcome of the pathological assessment. |
| 7.7 | Unit of Measurement | Standardized international units for recorded data. |
| 7.8 | Additional Notes | Any other relevant information, including methodological variations or contextual considerations. |

1. Table 8 Blood Biochemistry Analysis

| 8.1 | Observation Count | Number of phenotypic observations recorded. |
| --- | --- | --- |
| 8.2 | Biochemical Testing Protocol | Standardized methodology for assessing blood chemical composition, providing insights into metabolic, organ-specific, and systemic functions. |
| 8.3 | Testing Date | Date on which blood biochemical analysis was performed. |
| 8.4 | Measured Value | Recorded outcome of the biochemical analysis. |
| 8.5 | Unit of Measurement | Standardized international units for biochemical parameters. |
| 8.6 | Additional Notes | Any other relevant information, including methodological variations or contextual considerations. |

1. Table 9 Medical Imaging Data

| 9.1 | Observation Count | Number of phenotypic observations recorded. |
| --- | --- | --- |
| 9.2 | Imaging Data Format | Format of the acquired medical imaging data, including DICOM, NIfTI, JPEG, PNG, and TIFF. |
| 9.3 | Imaging Description | Overview of imaging modalities and techniques utilized to obtain visual representations of anatomical or functional features. |
| 9.4 | Imaging Date | Date on which the imaging procedure was conducted. |
| 9.5 | Measured Value | Recorded outcome of the imaging analysis. |
| 9.6 | Additional Notes | Any other relevant information, including methodological variations or contextual considerations. |

1. Table 10 Cerebrospinal Fluid (CSF) Analysis

| 10.1 | Observation Count | Number of phenotypic observations recorded. |
| --- | --- | --- |
| 10.2 | CSF Analysis Protocol | Standardized approach for the collection, processing, and analysis of cerebrospinal fluid to assess biochemical, cellular, and molecular characteristics. |
| 10.3 | Test Date | Date on which cerebrospinal fluid analysis was performed. |
| 10.4 | Measured Value | Recorded outcome of the cerebrospinal fluid analysis. |
| 10.5 | Unit of Measurement | Standardized international units for CSF parameters. |
| 10.6 | Additional Notes | Any other relevant information, including methodological variations or contextual considerations. |
